# Efficacy of 5% Dextrose Water Injection for Peripheral Entrapment Neuropathy: A Narrative Review

**DOI:** 10.3390/ijms222212358

**Published:** 2021-11-16

**Authors:** Yung-Tsan Wu, Chueh-Hung Wu, Jui-An Lin, Daniel Chiung-Jui Su, Chen-Yu Hung, Stanley K. H. Lam

**Affiliations:** 1 Department of Physical Medicine and Rehabilitation, Tri-Service General Hospital, School of Medicine, National Defense Medical Center, Taipei 11490, Taiwan; crwu98@gmail.com; 2 Integrated Pain Management Center, Tri-Service General Hospital, School of Medicine, National Defense Medical Center, Taipei 11490, Taiwan; 3 Department of Research and Development, School of Medicine, National Defense Medical Center, Taipei 11490, Taiwan; 4 Department of Physical Medicine and Rehabilitation, National Taiwan University Hospital Hsin-Chu Branch, Hsinchu 300, Taiwan; b88401062@ntu.edu.tw; 5 Department of Physical Medicine and Rehabilitation, College of Medicine, National Taiwan University, Taipei 100, Taiwan; 6 Department of Physical Medicine and Rehabilitation, National Taiwan University Hospital, Taipei 100, Taiwan; 7 Department of Anesthesiology, School of Medicine, College of Medicine, Taipei Medical University, Taipei 110, Taiwan; Juian.lin@tmu.edu.tw; 8 Department of Anesthesiology, Wan Fang Hospital, Taipei Medical University, Taipei 116, Taiwan; 9 Pain Research Center, Wan Fang Hospital, Taipei Medical University, Taipei 116, Taiwan; 10 Center for Regional Anesthesia and Pain Medicine, Wan Fang Hospital, Taipei Medical University, Taipei 116, Taiwan; 11 Department of Anesthesiology, School of Medicine, National Defense Medical Center, Taipei 11490, Taiwan; 12 Department of Physical Medicine and Rehabilitation, Chi-Mei Medical Center, Tainan 71004, Taiwan; dr.daniel@gmail.com; 13 Department of Physical Medicine and Rehabilitation, National Taiwan University Hospital, Beihu Branch, Taipei 10845, Taiwan; chenyu810@gmail.com; 14 The Hong Kong Institute of Musculoskeletal Medicine, Hong Kong, China; 15 Department of Family Medicine, The Chinese University of Hong Kong, Hong Kong, China; 16 Department of Family Medicine, The University of Hong Kong, Hong Kong, China

**Keywords:** entrapment nerve, 5% dextrose, ultrasound-guided, carpal tunnel syndrome, hydrodissection

## Abstract

Current non-surgical treatment for peripheral entrapment neuropathy is considered insignificant and unsustainable; thus, it is essential to find an alternative novel treatment. The technique of perineural injection therapy using 5% dextrose water has been progressively used to treat many peripheral entrapment neuropathies and has been proven to have outstanding effects in a few high-quality studies. Currently, the twentieth edition of Harrison’s Principles of Internal Medicine textbook recommends this novel injection therapy as an alternative local treatment for carpal tunnel syndrome (CTS). Hence, this novel approach has become the mainstream method for treating CTS, and other studies have revealed its clinical benefit for other peripheral entrapment neuropathies. In this narrative review, we aimed to provide an insight into this treatment method and summarize the current studies on cases of peripheral entrapment neuropathy treated by this method.

## 1. Introduction

Peripheral nerves are prone to entrapment in certain parts of the body, resulting in chronic hypoxia, inflammation, and other pathologies, which in turn will cause symptoms such as numbness, pain, paresthesia, and even muscle atrophy and weakness [1]. The increased pressure on the entrapment nerve leads to an interruption of nerve microcirculation, ischemia, impaired nerve conduction, dynamic decrement with adhesion, increased vascular permeability of the nerve, and interruption of axoplasmic flow with subsequent swelling of the nerve proximal and distal to the compression site [2,3,4]. Compression of the median nerve produces carpal tunnel syndrome (CTS), commonly in the wrist, compression of the ulnar nerve inside the elbow produces ulnar neuropathy in the elbow (UNE), compression of the radial nerve at the spiral groove produces radial nerve palsy, compression of the peroneal nerve under the outside of the knee produces peroneal neuropathy, medial ankle compression in the tibial nerves produces tarsal tunnel syndrome, compression of the lateral cutaneous nerves in the groin results in meralgia paresthetica, and toes produce Morton’s neuroma [5].

General treatment for peripheral entrapment neuropathy includes both non-surgical and surgical methods, which depends on the severity of symptoms. Non-surgical treatment includes avoiding persistent compression of the nerve, physical therapy, medications with nonsteroidal anti-inflammatory drugs (NSAIDs), local anesthetic, or corticosteroid injections. Although there are many conservative treatment methods, their effects are often insignificant and unsustainable. Surgical decompression is indicated if symptoms persist despite conservative management [5]. In the past few decades, corticosteroid injection has been one of the most commonly used non-surgical treatments for peripheral entrapment neuropathy, but previous studies have revealed its short-term efficacy [6,7]. The therapeutic mechanism of corticosteroids is based on their strong anti-inflammatory and analgesic effects that enable them to reduce the pressure of the compressive space and neuroinflammation. However, the onset of peripheral entrapment neuropathy is usually slow and chronic in nature; hence, it is considered a non-inflammatory ischemia-reperfusion degenerative neuropathy [1,8]. Therefore, it is assumed that the therapeutic effect of corticosteroids will be short-lived. Moreover, perineural injection of corticosteroids may cause direct neurotoxicity, resulting in widespread degeneration of axons and myelin, thus limiting its clinical usage [9]. Therefore, it is essential to find an alternative injectate for perineural injection to improve the success rate of non-surgical treatments.

## 2. Perineural Injection Therapy (PIT) with 5% Dextrose Water (D5W)

The concept of PIT using D5W to treat neuropathic pain was advocated by Dr. John Lyftogt in 2007, who revealed substantial pain control in patients with Achilles tendinopathy after this injection [10]. However, clinical evidence remains unclear, given the lack of well-designed clinical trials. Moreover, with the development of high-resolution ultrasound applications, ultrasound-guided injection can significantly promote accurate injection. Since 2017, few high-quality studies have proved the outstanding effect of ultrasound-guided PIT with D5W for CTS [11,12,13]. Currently, the twentieth edition of Harrison’s Principles of Internal Medicine textbook recommends this novel injection as an alternative local treatment for CTS [14]. Hence, this novel approach has become the mainstream method for treating CTS, and other studies have revealed its clinical benefit for other peripheral entrapment neuropathies. However, there are no current reviews dedicated to this novel injection therapy. Therefore, this narrative review article aimed to provide an insight into this treatment method and summarize the results of the recent studies on peripheral entrapment neuropathy treated by this method.

### 2.1. Mechanism

The effect of ultrasound-guided PIT with D5W on peripheral entrapment neuropathy can be classified as pharmacological, mechanical, and other possible neuroregenerative effects.

#### 2.1.1. Pharmacological Effect

Several characteristics of D5W make it suitable to be used for nerve injection. The osmolality of D5W is similar to that of normal saline and is painless than sterile water injection [15]. Prolotherapy injection is known to cause a local inflammatory cascade to recruit chemical mediators and growth factors to promote tissue regeneration and symptom relief in several chronic musculoskeletal pain diseases, especially degenerative disorders [16,17]. In contrast to hypertonic dextrose (>10%) injection of prolotherapy, D5W has an isotonic concentration and does not have a pro-inflammatory effect [18,19]. Moreover, D5W has been reported to be not harmful to nerves in animal and human studies [15,20]. However, the exact therapeutic mechanism of D5W on nerves is not clear, and the associated research is insufficient because no current research has directly investigated the effect of D5W on peripheral nerves. Nevertheless, based on previous studies, it can be inferred that there are potential sensorineural mechanisms behind such activity of D5W. Dextrose may indirectly downregulate capsaicin-sensitive receptors (transient receptor potential vanilloid receptor-1, TRPV1), which can be found on ligaments, tendons, joints, and peripheral nerves, and upregulation of TRPV1 is associated with neuropathic pain. The downregulation of TRPV1, in turn, impedes the discharge of substance P and calcitonin gene-related peptides, which are pro-nociceptive substances that contribute to neurogenic inflammation and neuropathic pain [21,22,23]. Further, nerves in a hypoglycemic environment induce the activation of nociceptive C fibers with increased noxious signal transduction, and elevated concentrations of extracellular dextrose could hyperpolarize C fibers [24]. A recent animal model revealed that dextrose could activate acid-sensing ion channel 1a (ASIC1a) and promote the release of substance P, which would trigger M-type potassium channels to modify the acid-sensing ion channel 3 (ASIC3)-positive afferents to relieve hyperalgesia [25]. However, further histological studies are needed to decipher the complete pharmacological mechanism behind the therapeutic effect of D5W on peripheral entrapment neuropathy.

#### 2.1.2. Mechanical Effect (Hydrodissection)

Hydrodissection (HD) during PIT is important for its therapeutic effect on peripheral entrapment neuropathy. HD can separate the compressed, injured nerve from the neighboring soft tissue to lower adhesion and chronic constriction injury, thus further increasing the blood flow and remobilizing the nerve for neuroregeneration [26,27,28]. Research published in 2019 first confirmed the clinical efficacy of HD for mild-to-moderate CTS in a double-blind trial, and the authors found that the HD effect could persist for 3 months in symptom relief and at least 6 months in reduction in cross-sectional area (CSA) of the median nerve after single nerve injection with 5 cc normal saline compared to placebo injection [27].

#### 2.1.3. Other Possible Neuroregenerative Effects

Although hypotonic dextrose (<10%) was considered to have no effect on inducing local inflammation for tissue regeneration, its possible neuroregenerative effect may exist due to subsequent improvements from pharmacological and mechanical effects or through other mechanisms currently unknown [29]. Based on the current series of research and clinical observations, mechanical HD could have a predominant role in the initial relieving of symptoms, followed by possible anti-neurogenic inflammation. Recently, Li et al. [13] reported a very long-term effect (mean of 1 to 3 years post-injection follow-up) of ultrasound-guided PIT using 10 mL D5W for CTS, which may result from possible subsequent neuroregenerative effects since only HD and pharmacological effects cannot explain such extreme long-term effect. However, further studies are required to prove this hypothesis.

### 2.2. Determined Factors for Hydrodissection Effect

The effect of HD depends on the guided method and the injectate content. Moreover, the effects of HD theoretically have volume and cumulative effects without direct evidence from current literature.

#### 2.2.1. Guided Method

Compared with blind injection, PIT in the use of ultrasound guidance is more precise, effective, and safe [30]. Chen et al. [31] showed that short-axis injection with a single 5 mL normal saline exhibited a more short-term HD effect compared to the long-axis injection for CTS, although its clinical significance is uncertain. This notable effect might be a result of higher HD between the subsynovial connective tissue (SSCT) and median nerve compared to long-axis injection, which mainly hydrodissects the median nerve from the flexor retinaculum as the fibrosis and adhesion of SSCT on the median nerve is an important part of the pathophysiology of CTS [32]. Hence, simultaneous HD above and below the entrapped nerve seems to be more effective than single HD either above or below the nerve. The more technically demanding long-axis approach superficial and deep to the median nerve has been highlighted by Lam et al. [33,34]. Further large-scale studies are needed to determine the most effective method for ultrasound-guided HD of a particular nerve in a specific location of the body.

#### 2.2.2. Injectate Content

The injectate content could also affect the therapeutic efficacy and duration of HD. Hyaluronic acid (HA), which has anti-adhesion effects, has been clinically applied for post-surgical adhesion [35,36]. Su et al. [37] reported that compared with HD with normal saline, HD with HA showed statistically significantly superior outcomes and functional scores at the second week post injection. This study also revealed sustained retention of injectate in the HA group one hour after injection, while normal saline was almost completely absorbed. Prolonged HA retention surrounding the nerve and its anti-adhesion characteristics may contribute to earlier symptom relief through lubricating adhesion, which improves MN mobility and decreases the pressure within the carpal tunnel [38,39,40]. Thus, the effect of HD may be dependent on the retention time of the injectate.

#### 2.2.3. Volume Effect

Although the recent studies have not established the volume effect of HD, previous studies indirectly supported the volume effect of HD in patients with CTS and corroborated our observation that a higher volume of injectate may have a more significant and lasting HD effect because it could more comprehensively separate nerves from areas of entrapment. Indeed, a study demonstrated insignificant alleviation of symptoms when only 1 mL normal saline blind injection was administered [41]. Partial symptom relief occurred one-week after 1 mL normal saline blind injection in merely 15% of patients [42]. Girlanda et al. [43] reported a noteworthy effect with improved motor action potential and nocturnal paresthesia, which continued up to 2 months after administering 15 mg normal saline injection (9 mg/cc). A similar finding was observed by Malahias that 33% of patients showed amelioration of symptoms 12 weeks after ultrasound-guided 2 mL normal saline injection [44]. Interestingly, when the volume of injectate was increased to 5 mL, a remarkable improvement in symptoms and CSA was observed 6 months post injection [11]. In addition to normal saline, a similar phenomenon was observed using corticosteroid and D5W injection. Evers et al. [45] revealed that a higher injection volume of corticosteroids could ensure better alleviation of symptoms with extended persistence of the effect. Recently, Lin et al. [46] found that HD with a larger volume (4 mL) of D5W provided improved efficacy in symptom relief and functional improvement of CTS than 1 and 2 mL D5W. However, further prospective researches to precisely compare the effect of different volume of normal saline injection for entrapment neuropathy are warranted to conclusively prove the volume effect of HD.

#### 2.2.4. Cumulative Effect

A recent retrospective study reported that an effective outcome was observed in 88.6% of patients with CTS after undergoing a mean of 2.2 times of PIT with 10 cc of D5W with a 1- to 3-year post-injection follow-up [13]. This extensive long-term effect was notably longer than that of a single PIT with 5 cc D5W reported in previous studies [11,12]. Moreover, CTS of different grades required different frequencies of PIT for an effective outcome (2.6 vs. 1.7 times in severe and mild CTS, respectively) [13]. Although the current study did not support the cumulative effect of HD, this retrospective study may indirectly confirm the cumulative effect of HD.

## 3. Search Method

Four databases (PubMed, EMBASE, Scopus, and Cochrane) were systematically searched up to 30 September 2021 for relevant literature, using combinations of keywords: ultrasound-guided, dextrose, injection, entrapment, nerve, CTS, cubital tunnel syndrome, UNE, radial nerve palsy, peroneal neuropathy, meralgia paresthetica, neuroma, and HD. We looked into studies assessing the efficacy and safety of ultrasound-guided PIT with D5W for peripheral entrapment neuropathy. Such assessments included studies on its clinical effects on pain intensity, clinical symptoms/function, physical performance, electrophysiological or ultrasound studies, etc. The inclusion criteria consisted of clinical studies including a retrospective study, letter, case reports/series investigating PIT with D5W for peripheral entrapment neuropathy published in the English language. The references of the articles were also searched to identify additional relevant publications. Any study that used a mixed injectate, corticosteroid, or vitamin with D5W for nerve injection was excluded.

## 4. Result

After thorough research of 50 potentially relevant articles, a total of 15 major publications on the subject were finally included in this review. The studies chosen included those on CTS, UNE, superficial radial nerve entrapment, radial nerve palsy, superficial peroneal nerve entrapment, meralgia paresthetica, posterior interosseous nerve entrapment (supinator syndrome), pronator teres syndrome, and deep nerve. There were five clinical trials (four studies on CTS and one on UNE) and two retrospective studies investigating CTS and deep nerves in the upper body and torso. The other eight clinical studies were case reports and letters. CTS is the most common type of entrapment neuropathy.

## 5. Clinical Trials

### 5.1. CTS

CTS is the most common peripheral entrapment neuropathy, accounting for 90% of the presentations. In 2017, Wu et al. [11] used a single ultrasound-guided PIT with 5 mL D5W for treating mild-to-moderate CTS in a randomized, double-blind study. They located the injection site at the proximal inlet of the carpal tunnel via the short-axis ulnar approach to simultaneously hydrodissect the SSCT and flexor retinaculum from the median nerve. The results showed significant improvement in symptoms, results of the electrophysiological study, and CSA of the nerve persisting for at least 6 months compared to normal saline injection. Moreover, they also ratified PIT with 5 mL D5W being superior to corticosteroid injection (3 mL triamcinolone (10 mg/mL) mixed with 2 mL normal saline) at four to six months post injection to reduce symptoms and disability [12]. Likewise, they retrospectively found that body height and sensory nerve conduction velocity of the median nerve were risk factors for poor outcomes after PIT with D5W in patients with mild-to-moderate CTS. Moreover, the sensory nerve conduction velocity of the median nerve was found to be an independent prognostic factor (odds ratio, 1.201) of poor outcome [47]. Nevertheless, the electrophysiological study presented a limited diagnosis of CTS with varied sensitivity (56% to 85%) and specificity (94% to 96%) [48,49]. For example, Martin-Gruber anastomoses may lead to misinterpretation or erroneous results during routine nerve conduction studies in patients with CTS [50]. Hence, underestimation of CTS severity may be attributed to the failure in proficient diagnosis. This would also be reflected as conflicting results in clinical trials on CTS. Recently, Lin et al. [46] designed a randomized, double-blinded, three-arm trial and ultrasound-guided PIT with D5W via a short-axis radial approach to simultaneously dissect the median nerve from the SSCT and flexor retinaculum. The results showed that the 4 mL D5W group had superior efficacy to 1 and 2 mL D5W in symptom relief and functional improvement at the 1, 4, and 12 weeks post injection. Moreover, their extended study revealed that PIT with a higher volume of D5W also enhanced nerve mobility and reduced the CSA of the median nerve [51].

The long-term effect of PIT with D5W on CTS was also satisfactory and safe, based on the latest study. Li et al. [13] retrospectively traced 185 patients with all grades of CTS who underwent ultrasound-guided PIT with 10 mL D5W using an initial short-axis injection with subsequent long-axis injection with a follow-up period of at least 1 year after the last injection (mean 1–3 years follow-up). The results revealed that 88.6% of the patients showed an effective outcome (symptom relief > 50% compared to baseline), while 62.7% of patients showed an excellent outcome (symptom relief > 70% compared to baseline) after a mean of 2.2 injections, and there were no complications in any of the patients. Moreover, only two patients ultimately underwent surgery after the failure of injection therapy to cure the condition. In addition, 80% of the patients (12 of 15 patients) had a surgical failure or post-surgery recurrence and had an effective outcome. Additionally, the outcome is considerably related to severity grade because the severe grade is associated with poor outcome compared to mild-to-moderate grade. A mean of 1.7, 2.4, and 2.6 injections was required to achieve an effective outcome in mild, moderate, and severe CTS, respectively [13].

### 5.2. Ulnar Neuropathy at the Elbow

UNE is the second most common entrapment neuropathy. In 2020, Chen et al. [52] reported that: PIT with corticosteroids (3 mL triamcinolone (10 mg/mL) mixed with 2 mL normal saline) or with 5 mL D5W both were effective in improving the symptoms of UNE, the outcomes of the electrophysiological study, and nerve CSA at the 6-month follow-up. However, the therapeutic effect of D5W in this study was not as effective as that reported in previous studies that used the same technique and injection volume to treat CTS [12]. Moreover, there was no significant difference in efficacy between the D5W and corticosteroid groups, which was also inconsistent with previous reports that showed D5W was more beneficial than corticosteroids for treating CTS 4–6 months post injection [12]. The different anatomy of the injected sites and the pathophysiology between CTS and UNE might explain this inconsistency. In addition to the carpal or cubital retinaculum in CTS and UNE, respectively, only one flexor carpi ulnaris tendon surrounds the ulnar nerve, while several tendons (flexor pollicis longus with eight flexor digitorum superficialis/profundus) surround the median nerve. Hence, the tissue covering the ulnar nerve at the elbow is very thin, without structurally containing boundaries such as the median nerve within the carpal tunnel. Therefore, the injectate may infiltrate into other layers during PIT for UNE, even with ultrasound guidance, unlike the median nerve, which is located inside the carpal tunnel. Compared to UNE, CTS involves a more intense density of perineural space and adhesion with surrounding soft tissues; hence, the HD effect could be greater in CTS than in UNE because HD mainly relieves pressure and adhesion of nerves [27]. This could be confirmed by the feeling of greater tension during HD between the median nerve and SSCT in CTS than that below the ulnar nerve in UNE.

### 5.3. Deep Nervous Structure

Lam et al. [28] reviewed the outcomes of 100 HD treatments in 26 cases, including cervical root compression, thoracic outlet syndrome, cervicogenic headache with a mean neuropathic pain duration of 16 months and a mean numeric pain rating scale (NPRS) of 8.3. They dissected the stellate ganglion, brachial plexus, and cervical nerve roots, depending on individual neuropathic characteristics. The mean percentage of analgesia during each HD was 88.1% ± 9.8%, and pretreatment NPRS improved from 8.3 ± 1.3 to 1.9 ± 0.92 months after the last treatment after mean 3.8 ± 2.6 treatments over 9.7 ± 7.8 months follow-up. Moreover, all patients had more than 50% pain relief, and half had more than 75% relief. Lam et al.’s results confirm the analgesic effect of D5W injection and also suggest that HD using D5W provides cumulative pain reduction (Table 1).

## 6. Case Report

### 6.1. Superficial Radial Nerve Entrapment

Chang et al. [53] reported a 73-year-old woman who had persistent tingling sensation over the radial wrist after significant relief of chronic De Quervain’s disease after corticosteroid injection. Hence, they used two ultrasound-guided PITs with 2 mL D5W to treat the entrapment of the superficial radial nerve at the wrist with the inspiratory effect of diminished paresthesia. Wei et al. [54] reported a 42-year-old woman with superficial radial nerve entrapment (numbness with electric burning sensation) in the last two years after surgery with fixed plates under a diagnosis of fracture at the left ulna and radius. They performed ultrasound-guided PIT with D5W at one-month intervals for a total of six injections, and complete symptom relief was noted after injection.

### 6.2. Radial Nerve Palsy

Chen et al. [55] studied a 62-year-old woman with a drop finger and wrist under the diagnosis of radial nerve palsy due to sleep with the arm compressed against her body. The patient showed outstanding improvement after two ultrasound-guided PITs with 15 mL of D5W. Not only noteworthy improvements of sensory and motor functions but also improved electrophysiological study were observed after PIT injection.

Su et al. [56] reported two cases of radial palsy after a humeral shaft fracture successfully treated with ultrasound-guided PIT with D5W. The first case was a 31-year-old man who developed left radial palsy after removal of the plate 14 months after surgery. Due to sustained wrist drop for 2 months, the patient received PIT with 15 mL D5W thrice at two-week intervals. Three months post injection, his neuropathic pain completely resolved, and the strength of the wrist and finger extensors noticeably improved from 1 to 4+ and 1 to 4, respectively. Moreover, the follow-up electrophysiological study showed improvement. In addition, the hardness of the surrounding tissue and radial nerve measured by shear-wave elastography significantly decreased compared to pre-injection, although follow-up ultrasonography showed persistent swelling of the radial nerve. Twenty-one months after PIT, the patient regained full motor function with no numbness. The second case involved a 43-year-old man who had sustained a postoperative radial nerve palsy for one year because the radial nerve was compressed underneath the plate. One year after removal of the plate with neurolysis of the radial nerve, his motor function returned to normal. However, severe tenderness and allodynia were present over the cutaneous innervation areas of the posterior cutaneous nerve of the forearm and the superficial radial nerve. After a total of 6 PITs with 60 mL D5W at two-week intervals, the neuropathic pain on the forearm improved. Two years after the last PIT, there was no numbness or allodynia.

### 6.3. Supinator Syndrome

Chen et al. [57] reported a 58-year-old man with drop finger for 3–4 months (the muscle strength of the left second and third fingers was 2, and that of the fourth and fifth fingers was 0) diagnosed with supinator syndrome. They performed a single ultrasound-guided PIT with 5 mL D5W to dissect the posterior interosseous nerve. At 1.5 months of follow-up, the strengths of the left second and third fingers had improved by at least one grade, and trace movement was noted in the left fourth and fifth fingers.

### 6.4. Meralgia Paresthetica

Su et al. [58] studied a 35-year-old woman with chronic meralgia paresthetica having tingling sensation, pain, or numbness in the anterolateral region of the thigh for 20 years. After receiving 7 ultrasound-guided PITs with 10 D5W on the lateral femoral cutaneous nerve within 2 months, she experienced significant symptom relief, and clear improvement was observed in the results of electrophysiologic study, accompanied by decreased swelling of the nerve evaluated by sonography at 6 months post injection.

### 6.5. Superficial Peroneal Nerve Entrapment

Chiang et al. [59] reported that a 58-year-old woman had right anterolateral shin pain for 4–5 months without local trauma. Ultrasound revealed entrapment of the superficial peroneal nerve between the peroneus brevis and extensor digitorum longus muscles. They performed a single ultrasound-guided PIT with 5 mL D5W on the superficial peroneal nerve, and the patient reported remarkable pain alleviation 2 weeks post injection. Complete alleviation of symptoms was also observed at six months follow-up.

### 6.6. Pronator Teres Syndrome

Chang et al. [60] reported a 25-year-old man who complained of pain and weakness in the right first and second finger flexion (grade 4 on the manual muscle testing grading system) after he strained his right forearm for 6 months. There was no sensory deficit in the right palm and fingers. Under the diagnosis of pronator teres syndrome, they performed five ultrasound-guided PITs with 10 mL D5W (at 2 weeks interval) to dissect the median nerve. The patient showed more than 50% strength improvement in his right thumb flexion (muscle strength grading improved to grade 4+) after five injections (Table 2).

## 7. Clinical Pearls

- PIT with D5W using the ultrasound-guided short-axis approach to simultaneously dissect below and above the entrapment nerve was recommended. Furthermore, using the short-axis approach to initially expand the perineural space followed by the long-axis injection could be more comprehensive and effective than only short-axis injection alone. The short-axis approach is simply repeated by pivoting the transducer and the HD direction to the proximal and thence to the distal part of the most entrapped part of the nerve, using the same needle entry point [33,34];
- Although the optimal dosage and frequency of PIT with D5W for entrapment nerve remain unknown, 5 to 20 cc D5W per injection administered twice or thrice is suggested based on the entrapment site and severity. The recommended injection interval is 1–4 weeks, according to the prognosis;
- During injection, the patients showed increased numbness and tightness caused by the volume effect or HD-related dragging effect on the nerve. The numbness/tightness may persist for several minutes and progressively decline within one hour;
- In cases where the patient is afraid of pain, a skin numbing with local anesthesia at the puncture site is suggested. Local anesthesia deeply into the nerve is not recommended because of the adverse effects of temporary nerve paralysis and possible neural toxicity [61].

## 8. Conclusions

PIT with D5W is a novel and effective approach for CTS based on the current series of high-quality clinical studies. This injection may also be an effective method for other peripheral entrapment neuropathies, but there is a lack of compelling data to support its effectiveness. Moreover, the current literature is mainly derived from a few studies, each conducted within a particular country, which may produce a population and geographic bias. Likewise, there are many questions regarding this technique that are yet to be clarified, such as the definite pathophysiological effect of D5W on nerves, whether there is a cumulative effect of PIT with D5W and the optimal dosage and frequency of PIT with D5W for entrapment neuropathy. In addition, the HD approach was more effective. Therefore, further studies need to be conducted to develop an optimum treatment strategy for peripheral entrapment neuropathy.

## Figures and Tables

**Table 1:** Summary of included clinical studies.

Author (Year)	Study Design	Inclusion Criteria	Injection Method	Participant Characteristics					Outcome Measurements	Follow-Up	Safety Outcome
			Treatment Allocation	Disease	Sample Size	Mean Age (Years)	Female (%)	Symptom Duration (Months)			
Wu (2017)	Randomized, double-blind trial	Clinical + EDS	Single UG injection (ulnar S-I below and above MN)	Mild to moderate CTS	30/30 (Wrists, cases/controls)	58.4/58.1 (Cases/controls)	86.7/80 (Cases/ controls)	44.5/44.4 (Cases/controls)	VAS, BCTQ, EDS, CSA of MN, Global assessment of treatment results	6 months	No AE reported
			5 mL D5W vs. 5 mL NS								
Lam (2018)	Retrospective study	Clinical	Mean 3.8 UG injection with 20–30 mL D5W (S-O then L-I above and below stellate ganglion, brachial plexus, cervical nerve roots)	Cervical root compression, Thoracic outlet syndrome, Cervicogenic headache	26	NA	NA	46 months	Numeric Pain Rating Scale	9.7 ± 7.8 months	No AE reported
Wu (2018)	Randomized, double-blind trial	Clinical + EDS	Single UG injection (ulnar S-I below and above MN)	Mild to moderate CTS	27/27 (Wrists, cases/controls)	58.6/54.3 (Cases/controls)	81.4/77.7 (Cases/controls)	46.8/45.6 (Cases/controls)	VAS, BCTQ, EDS, CSA of MN, global assessment of treatment results	6 months	No AE reported
			5 mL D5W vs. 3 mL triamcinolone (10 mg/mL) + 2 mL NS								
Chen (2020)	Randomized, double-blind trial	Clinical + EDS	Single UG injection (S-I below and above UN)	Mild to moderate UNE	17/16 (Elbows, cases/controls)	55.5/56.5 (Cases/controls)	70.6/62.5 (Cases/ controls)	44.4/41.6 (Cases/controls)	VAS, DASH, EDS, CSA of UN, global assessment of treatment results	6 months	No AE reported
			5 mL D5W vs. 3 mL triamcinolone (10 mg/mL) + 2 mL NS								
Lin (2020)	Randomized, double-blinded, three-arm trial	Clinical + EDS	Single UG injection (radial S-I below and above MN)	CTS (NR grade)	21/21/21 (Wrists, cases/controls)	58.4/55.2/60.3 (Cases/controls)	95.2/81/81 (Cases/ controls)	54.4/20.6/49.8 (Cases/controls)	VAS, BCTQ, Q-DASH, EDS, and CSA of MN	6 months	No AE reported
			4 mL D5W Vs. 2 mL D5W V. 1 mL D5W								
Lin (2021)	Randomized, double-blinded, three-arm trial	Clinical + EDS	Single UG injection (radial S-I below and above MN)	CTS (NR grade)	17/14/14 (Wrists, cases/controls)	56.9/52.9/59.2 (Cases/controls)	94.1/85.7/85.7 (Cases/ controls)	66/21.9/58.4 (Cases/controls)	mobility, shear-wave elastography CSA of MN, VAS, BCTQ	6 months	NR
			4 mL D5W Vs. 2 mL D5W Vs. 1 mL D5W								
Li (2021)	Retrospective study	Clinical + EDS	Mean 2.2 UG injections with 10 mL D5W (ulnar S-I below and above MN + L-I from proximal to distal)	All grade CTS	185 (wrists) No control	55.4	65.4	30.8	VAS Surgical rate	At least 1 year (1–3 years) post injection (mean 15.8 months)	No AE reported

UG: ultrasound-guided; D5W: 5% dextrose water; NS: normal saline; CTS: carpal tunnel syndrome; VAS: visual analog scale; NR: not reported; AE: adverse effect; BCTQ: Boston carpal tunnel syndrome questionnaire; EDS: electrodiagnostic study; CSA: cross-sectional area; MN: median nerve; UN: ulnar nerve; 2PD: two-point discrimination; Q-DASH: Quick Disability of the Arm, Shoulder and Hand score; S-I: short-axis in-plane; S-O: radial short-axis out-plane; L-I: long-axis in-plane; UNE: ulnar neuropathy at elbow.

**Table 2:** Summary of included case reports.

Author (Year)	Injection Method	Participant Characteristics			Outcome Measurements	Follow-Up
		Diagnosis	Age (Year)/Sex	Symptom Duration		
Chang (2015)	Two UG injections with 2 mL D5W (S-I above superfical radial nerve)	Superficial radial nerve entrapment	73/Female	6 months	Symptom	Post 2 injections but NR of injection interval
Chen (2018)	Two UG injections with 15 mL D5W (S-I below and above radial nerve + L-I from distal to proximal)	Radial nerve palsy	62/Female	2 months	Symptom, EDS, CSA of nerve	3 months
Chen (2018)	Single UG injection with 5 mL D5W (S-I above posterior interosseous nerve)	Supinator syndrome	58/Male	3–4 months	Muscle strengths	1.5 months
Su (2020)	Seven UG injections with 10 mL D5W (S-I below and above lateral femoral cutaneous nerve + L-I from distal to proximal)	Meralgia paresthetica	35/Female	20 years	Symptom, EDS, CSA of nerve	6 months
Su (2020)	Three UG injections with 15 mL D5W (S-I above and below radial nerve at two-week intervals)	Radial nerve palsy	31/Male	2 months	Symptom, EDS, CSA of nerve, shear-wave elastography	21 months
	Six UG injections with 60 mL D5W (S-I above and below radial nerve at two-week intervals)		43/Male	2 years		2 years
Wei (2020)	Six UG injections with D5W (unknown dosage) (S-I above superfical radial nerve at one-month intervals)	Superfical radial nerve entrapment	42/Female	2 years	Symptom	6 months
Chiang (2020)	Single UG injection with 5 mL D5W (S-I above superfical peroneal nerve)	Superfical peroneal nerve entrapment	58/Female	4–5 months	Symptom	6 months
Chang (2021)	Five UG injections with 10 mL D5W (L-I above median nerve at two-week intervals)	Pronator teres syndrome	25/Male	6 months	Symptom	Post 5 injections

UG: ultrasound-guided; D5W: 5% dextrose water; NR: not reported; EDS: electrodiagnostic study; CSA: cross-sectional area; S-I: short-axis in-plane; L-I: long-axis in-plane.

## Data Availability

Not applicable.
